# Pharmaceutical Industry Payments to Patient Organizations in Poland: Analysis of the Patterns, Evolution, and Structure of Connections

**DOI:** 10.1177/27551938241305995

**Published:** 2024-12-26

**Authors:** Marta Makowska, Shai Mulinari, Piotr Ozieranski

**Affiliations:** 1 Department of Economic Psychology, Kozminski University, Warsaw, Poland; 2 Department of Sociology, 5193 Lund University, Lund, Sweden; 3 Department of Social and Policy Sciences, 1555 University of Bath, Bath, UK

**Keywords:** Conflict of interests, transparency, NGO funding, public trust, pharmaceuticals

## Abstract

Drug company funding can create conflicts of interest that compromise the integrity of patient organizations, a problem studied primarily in Western Europe and North America. To address this research gap, we conducted a case study in Poland, a Central European country. Between 2012 and 2020, 33 companies reported payments worth €13 729 644 to 273 patient organizations in Poland. The funding was highly concentrated, with the top ten recipients amassing 46.2 percent of the total amount. Cancer patient organizations were the primary recipients, receiving 37.5 percent. The funding focused on patient organizations’ educational activities, constituting 40.4 percent of the total. For the ten companies reporting payments consistently from 2012 to 2020, we detected an increase in both the value of individual payments and the overall value of the funding. Additionally, some patient organizations formed exclusive, or nearly exclusive, ties with single companies. Overall, our study reveals important similarities between Poland and Western countries in the reported distribution of drug company payments to patient organizations. It also highlights priority areas for further research, including the evolution and structure of the financial connections.

Partnerships between patient organizations and pharmaceutical companies are intensely debated in international health policy. Some argue that they empower patient organizations, as drug companies offer much-needed funding for patient care, disease awareness, and advocacy.^1,2^ However, critics argue that when patient organizations receive funding from companies, it can create conflicts of interest. This money might influence patient organizations to support companies’ goals instead of pursuing their own independent objectives, which risks turning them into allies for companies’ lobbying and marketing efforts.^1–7^

Available country case studies^8–14^ and systematic reviews^15,16^ show that financial ties between patient organizations and pharmaceutical companies are widespread^8–14^ and that a handful of donors and recipients account for most of the monetary transactions.^8,9,17,18^ Industry funding usually prioritizes high-profile conditions, particularly cancer,^7–9,17–19^ with mounting evidence linking funding to drug portfolios, including newly launched or top-selling products.^17,20^ Similarly, patient organizations with specific organizational and activity profiles are prioritized. For example, in the United Kingdom, where these elements have been studied extensively, most of the funding goes to patient organizations officially registered as charities or associations, with multiple areas of activity ranging from patient support to advocacy.^9^

Countries vary in the overall number of donors and recipients, the value of funding,^9,11,17,19^ and the extent of patient organizations’ reliance on industry income for their operations.^3,8,12,18,20–22^ Companies’ funding priorities also seem to differ between countries. For example, in the United Kingdom,^9^ industries prioritize funding of patient organizations’ research activities, while in Sweden^15^ communication initiatives take precedence. By contrast, in Australia,^7^ funding primarily supports information materials and dissemination. Further, funding does not necessarily correlate with a country's population size, as Swedish and Danish patient organizations receive similar volumes from the industry.^19^ Variation also exists in the industry's prioritizing advocacy funding for diseases other than cancer. For instance, endocrine, nutritional and metabolic diseases, and infectious and parasitic diseases collectively received 19 percent and 17 percent of the funding in Sweden and the United Kingdom, respectively.^9,17^ However, key conditions from these disease areas received little funding in Australia, with only three percent allocated to diabetes and two percent to HIV/AIDS.^7^

A significant limitation of this body of international research is its focus on high-income countries,^15^ primarily Western European nations such as Spain,^23^ Ireland,^24^ the Netherlands,^25^ the United Kingdom,^8–10^ the Nordics,^11,17,19,21^ and North America (the United States^3,12,20,26,27^ and Canada^5,13,14^) and Australia.^1,2,28^ Consequently, the identified patterns of financial ties may reflect the specific Western context of patient groups and their relationships with other policy actors^29^ or unique institutional settings, such as the frameworks for patient group involvement in drug appraisals in the United Kingdom and Canada.^5,10,13,29^

Furthermore, few studies have examined the structure of relationships, including the number of donors per recipient.^7–9,17^ This is a significant research gap since more exclusive relationships may pose a higher risk of industry capture of patient organizations.^4^ Industry, too, acknowledges these risks, with the European Federation of Pharmaceutical Industries and Associations (EFPIA) Code of Practice encouraging multiple sponsors.^30^

Finally, funding trends have received limited attention, with most studies aggregating longitudinal data^3,7^ or comparing only basic characteristics of funding distribution over time.^7,9,17^ Funding dynamics can evolve differently, such as with the increasing trend in overall funding identified in the United Kingdom^9^ but decreasing in Sweden.^17^ Furthermore, sustained funding of specific organizations over time suggests stable “business relationships”,^2^ indicative of the formation of a “patient - industry complex” associated with a higher risk of capture.^18^ However, sporadic relationships might reflect weaker ties between companies and patient organizations and a lower risk of capture even when some payments are occasionally large.

In seeking to address these gaps in research, we focused on Poland, a Central European country with the sixth largest pharmaceutical market in Europe.^31^ Poland has transitioned from the communist Semashko-style health system (a single-payer health care system where health care is free for everyone, funded by the government) towards a version of the Bismarck-type insurance-based system. Unlike the U.S. insurance industry, Bismarck-type health insurance plans are nonprofit and must cover everyone.^32^

We seek to, first, analyze the pattern of drug company payments to patient organizations and evaluate the key similarities and differences with findings from Western Europe; second, contribute to filling the research gaps concerning the structure and trends in financial relationships between drug companies and patient organizations.

## Patient Organizations and the Pharmaceutical Industry in Poland: the Institutional Context

Until the fall of communism in 1989, the state heavily constrained possibilities to engage in health activism in Poland,^33^ with patient organizations comprising only approximately two percent of the otherwise small sector of Polish nongovernmental organizations.^34^ With the transition to a market economy, the number of patient organizations rose significantly,^34^ reflecting the broader trend of civil society expansion in the late 1990s and early 2000s, facilitated by foreign aid, institutional reforms,^35^ and dedicated funding associated with the accession to the European Union in 2004.^36^

The current number of patient organizations in Poland is estimated at 1000,^34^ which closely aligns with Finland (130 patient organizations), considering the difference in the population size (Poland, about 38 million; Finland, about 5 million^21^). While some patient organizations are informal support groups, others have a “legal personality” and, as such, are registered in the National Court Register (*Krajowy Rejestr Sądowy*; KRS).^37^ There are penalties for registered patient groups if they do not submit the required documents on time to the KRS.^37^ Therefore, the data about the organizations in the KRS are likely to be reliable. Specifically, registered patient organizations must document their activities by submitting up-to-date information to the KRS, including their name, address, scope, nature of activity, management board composition, and annual financial reports.^37^ These financial reports include a balance sheet, income statement, and supplementary information, but the KRS does not require specifying donors, such as pharmaceutical companies.^38^ The main legal forms for registered patient organizations are foundations and associations.^34^ Foundations are established by a founder or the board, with at least €220 in initial capital, and should raise funds for socially relevant goals. On the other hand, associations are groups pursuing common interests and goals, requiring no initial capital but charging membership fees (see Online Appendix 1).

Polish patient organizations’ main reported priorities align with the access-oriented consumerism dominant in the West,^39,64^ seeking to broaden the availability of new therapies primarily within state-funded reimbursement plans.^40,41^ Like in the West, access-oriented consumerism in Poland involves joint advocacy with drug companies, especially concerning health technology assessment and drug reimbursement,^42–44^ including in commercially highly profitable areas like cancer, diabetes, and rare disorders.^42,45^

Yet, unlike in countries such as the United Kingdom or the Netherlands,^46^ no special provisions exist for patient organizations’ engagement in health policymaking.^29^ Previously, patient organizations in Poland might have displayed relatively higher reliance on drug company funding (and other resources, such as expertise), resulting from limited government funding and a heavily underfunded health system during a radical economic transition.^32,47^ Nevertheless, more recently, government support has been declining in some Western countries,^6^ forcing patient organizations to rely more on industry support. Indeed, in Poland, patient organizations may in some ways enjoy an advantageous situation as industry (and public) funding could be partially offset by the increasingly popular tax earmarking,^48^ allowing taxpayers to donate 1.5 percent of their income tax to nongovernmental entities registered as public benefit organizations in the KRS.^49^ However, achieving and maintaining the public benefit status may not be practical or desirable for all organizations, as it entails additional reporting such as publishing updates on activities and financial reports.^50^

Like elsewhere in Europe, Poland has no legislation governing transparency in partnerships between drug companies and patient organizations. Instead, these relationships are governed through industry self-regulation based on rules drawn up by the European drug industry trade group, EFPIA,^51^ and adopted and implemented by the local Polish drug industry trade group Infarma^9,52^ via its “Code of Good Practices,” or the “Infarma Code” for short. Consistent with the minimum standards set by EFPIA, the Infarma Code stipulates that trade group members must disclose *a*) the names of the patient organizations with whom they work, *b*) the nature of the assistance provided or services rendered, and *c*) the monetary value of the support or services, or a description of the benefits in kind. The disclosure reports should be published annually on each company's website and kept there for at least three years. In addition, a few non-Infarma members also report their payments to patient groups; for example, the foremost Polish generic company Polpharma discloses payments because it is a member of the Polish Association of Pharma Industry Employers—Domestic Drug Manufactures (PZPPF^53^). In contrast to Infarma members, PZPPF members do not disclose their payments to patient organizations individually or even highlight which recipients are patient organizations. It would require forensic analysis to determine which recipients are indeed patient organizations.

One previous study from Poland^54^ identified that even Infarma members’ reporting of payments had not been fully transparent, comparable to the problems well documented in the West, including the underreporting of payments^9,25,35,52,55–57^ and incomplete information about the purpose of funding, its value and recipients, in apparent violation of the industry's own self-regulatory rules.^8,14,20,27,52,58^ Nevertheless, the broader context of health care governance suggests that companies in Poland may be more likely to breach transparency and other self-regulatory provisions compared to the same companies in the West.^42^ For example, as of 2022, Poland ranked 45th out of 180 countries in the Corruption Perceptions Index,^59^ with health care highlighted as the second most corrupt sector.^45^ Recalling some informal norms used by people and organizations for navigating the communist economy of shortages,^60^ unethical practices such as bribery are recognized as standard behavior, for example, in securing privileged health care access.^45^ Additionally, there is strong skepticism that transparency can ensure ethical conduct in Poland; concerns are expressed within the medical profession about unintended consequences of transparency, such as patients avoiding physicians with known ties to industry.^55^

## Methods

### Payment Data

Our primary data source was payment disclosures published online by companies that subscribe to the Infarma Code. We collected 208 disclosure reports between June 2020 and November 2021. All available disclosure reports were initially collected from individual companies’ websites, as no central database covers the period from 2008 to 2020. However, we excluded reports from 2008 to 2011 because only 19 reports were available from those years. We excluded six further reports that contained no payment information. Overall, we analyzed 183 reports (see Online Appendix 2).

The payment data in the 183 reports were extracted manually into a single Excel spreadsheet, with all payments adjusted using the National Bank of Poland's consumer price inflation index.^56^ Payments reported in Polish *złoty* were converted to euros using the average yearly exchange rates published by the European Central Bank.^57^ We standardized the names of patient organizations and aggregated organizations with regional branches. We used a separate variable to connect payments to their location in Poland's different administrative regions called *voivodeships*.

We extracted 2838 payments reported between 2012 and 2020, with a total reported value of €14 700 546. We excluded 242 payments (€970 902), including those not made to patient organizations (n = 118, €444 949), made to recipients who could not be identified through internet searching (n = 87, €311 630), made to recipients whose nature was unclear (n = 29, €177 896), and made to patient organizations outside of Poland (n = 8, €36 428; see Online Appendix 3). Payments with missing or incomplete values (0.3%; n = 8) were retained as zero values.

### Coding and Organizational Characteristics

Following a previous Australian study,^7^ we coded *patient organization disease areas* using an inductive categorization (see Online Appendix 4). This enabled us to better reflect relevant disease areas in Poland (e.g., HIV/AIDS) than using the ICD-10 codes applied in some previous studies.^17–19^

Our categorization of *payment goals* was also inductive, again drawing on the aforementioned Australian study.^7^ We initially considered a deductive categorization based on earlier U.K.^9^ and Swedish research,^17^ but it would have resulted in significant data loss, as several areas of activity receiving considerable funding could not be meaningfully covered by the existing codes, such as COVID-19-related work or improving access to medical services (see Online Appendix 5).

To maximize consistency, we coded *the aims of patient organizations* using the organizations’ official registration information in the KRS rather than taking the information from their websites. We developed an inductive classification (see Online Appendix 6) to capture aims unique to Poland, allowing multiple aims for each organization. This approach to coding avoided a sizeable category of patient organizations with multiple aims identified in U.K. research.^9,18^

We utilized the KRS data to establish patient organizations’ *legal status* (foundation, association, or federation), *location* (one of the 16 voivodeships), and whether they were public benefit organizations.

One researcher on our team conducted the data collection, extraction, and coding in consultation with the entire team. Each company website was checked three times, and the accuracy of data extraction was checked twice for each report.

No ethics approval was needed as the research involved organizational-level, publicly available data.

## Analysis

We analyzed *payment patterns* descriptively, including the number, value, median, and interquartile range of payments for each year and all years combined. We also assessed *payment concentration*, determining the funding share each year and all years combined from the top ten payments, donors, and recipients and the share of funding from the top donors and recipients. Additionally, we analyzed the share of funding for the ten most funded disease areas. We tabulated payment goals broken down by recipients, donors, and the value of payments.

To explore how industry funding *varied across patient organizational characteristics*, we analyzed the number, value, and median of the payments to patient organizations with different aims. Further, we analyzed the payment medians according to patient organizations’ legal status, location, and status as public benefit organizations (yes or no). We used the Chi-square test and the Kruskal-Wallis test with Bonferroni correction for post-hoc pairwise comparisons to assess statistical significance, with *p *< .05.

In analyzing *payment trends*, we considered the ten companies (Amgen, AstraZeneca, Biogen, Chiesi, GlaxoSmithKline, Merck, Janssen-Cilag, Pfizer, Roche, Takeda) that disclosed payments consistently throughout the observation period (2012 to 2020). These companies met and exceeded the EFPIA's minimum three-year data retention period in the public domain. For others, it was unclear whether they did not make payments in specific years, made payments but removed the disclosure reports, or made payments without publishing the reports.^9,52^ We compared the medians, number, and value of the payments from those ten companies in different years and the number of organizations receiving their payments. We assessed the trend in the medians using Kendall's tau-b correlation coefficient.

We examined the *structure of connections* between companies and patient organizations by analyzing the number of donors per patient organization over the years. We also calculated the number of years of collaboration between patient organizations and companies, considering separately only the ten companies for which we had reports for all years.

IBM SPSS version 28 was used for all analyses.

## Results

### Payment Patterns

#### Donors and Payments

Between 2012 and 2020, 33 companies reported 2588 payments worth €13 729 644 (see Table 1). Payments were highly concentrated, with the top ten donors (see Online Appendix 7) responsible for 79.1 percent (€10 859 432) and the top ten payments for 10.1 percent of the total (€1 389 263). Most payments fell within the €1000–€5000 range, accounting for 56.9 percent of the total (n = 1473). Larger payments exceeding €5000 constituted 28.5 percent (n = 738) of the payments, while smaller payments, less than €1000, constituted 14.6 percent (n = 377) of the total.

**Table 1.** Distribution of Reported Drug Company Payments to Patient Organizations in Poland, 2012–2020.

	2012	2013	2014	2015	2016	2017	2018	2019	2020	Total
No. of payments	190	228	225	228	243	286	356	397	435	2588
Value of payments, Euro	775 225	872 703	1 059 962	1 624 111	1 446 015	1 555 437	2 012 723	1 964 197	2 419 271	13 729 644
Median (IQR), Euro	1668 (923 to 3753)	2441 (1221 to 4563)	2471 (1236 to 4942)	3356 (1746 to 6234)	3576 (2122 to 7152)	3572 (1683 to 7144)	3582 (1571 to 7124)	3528 (1646 to 5880)	3376 (2251 to 6752)	3105 (1430 to 5953)
Number of companies with at least one valid payment reported	12	14	14	16	20	24	27	28	25	33*
No. of patient organizations	80	80	80	90	95	98	92	95	111	273*
*Share of funding by top 10*										
Payments	328 184 (42.3%)	216 744 (24.8%)	351 599 (33.2%)	765 262 (47.1%)	409 434 (28.3%)	372 735 (24%)	431 621 (21.4%)	386 424 (19.7%)	377 119 (15.6%)	1 389 263** (10.1%)
Donors	769 662 (99.3%)	836 289 (95.8%)	1 025 929 (96.8%)	1 549 441 (95.4%)	1 338 985 (92.6%)	1 356 120 (87.2%)	1 593 065 (79.1%)	1 445 622 (73.6%)	2 028 189 (83.8%)	10 859 432** (79.1%)
Recipients	489 559 (63.1%)	512 548 (58.7%)	644 574 (60.8%)	1 065 167 (65.6%)	792 195 (54.8%)	828 536 (53.3%)	1 155 551 (57.4%)	1 024 181 (52.1%)	1 339 078 (55.3%)	6 338 134** (46.2%)
*Share of funding by single top donor and recipient*										
Top donor – value of payments / number of payments/ number of recipients	197 005 (Roche) N = 84 payments/ 43 recipients	249 512 (Biogen) N = 42/ 5 recipients	296 450 (Pfizer) N = 16/ 12 recipients	682 180 (Pfizer) N = 17/ 15 recipients	275 583 (Janssen – Cilag) N = 32/ 19 recipients	299 263 (Pfizer) N = 16/ 11 recipients	294 485 (Janssen – Cilag) N = 48/ 24 recipients	216 365 (Sanofi) N = 31/ 15 recipients	397 427 (Sanofi) N = 62/ 31 recipients	1 989 651 (Roche) N = 531/101 recipients
Top recipient - value of payments / number of donors	151 657 2 donors	138 449 3 donors	138 878 1 donor	549 809 1 donors	211 847 6 donors	209 974 1 donors	248 884 7 donors	142 580 9 donors	3 169 147 11 donors	1 123 812*** 1 donor

* The total does not add up as the same companies/organizations are found in multiple cells.

** Refers to top 10 payments/donors/recipients 2012–2020.

*** Refers to the top recipient of payments 2012–2020.

#### Recipient Characteristics

A total of 273 patient organizations received payments. The funding was highly concentrated, with the top ten recipients receiving 46.2 percent (€6 338 133) of the total (see Table 1 and Online Appendix 8). Cancer patient organizations were the largest recipients, receiving 37.5 percent of the funding (see Table 2). They were followed by organizations focused on neurological conditions (18.4%) and bacterial diseases/vaccination (9.7%). Notably, organizations focused on bacterial diseases/vaccination received only 19 payments, out of which twelve, worth €1 123 812 (representing 10.1% of all reported payments), were made by Pfizer to a single organization focused on meningococcal and pneumococcal bacterial diseases.

**Table 2.** Ten Disease Areas with the Highest Value of Drug Company Funding in Poland, 2012–2020.

Disease area	Value of payments, Euro (%)	No. of payments
Oncology	5 151 699 (37.5%)	1003
Neurology	2 523 405 (18.4%)	497
Bacterial diseases (meningococci, pneumococci/infectious/promotion of vaccination)	1 331 279 (9.7%)	19
Rare diseases	993 229 (7.2%)	164
Diabetology	326 928 (2.4%)	64
HIV/AIDS	320 546 (2.3%)	87
Pulmonology	318 822 (2.3%)	68
Urology	310 805 (2.3%)	65
Hepatology	287 513 (2.1%)	89
Dermatology	246 441 (1.8%)	83
**TOTAL**	**11** **810** **666 (86.0%)**	**2139 (82.6%)**

The primary reported focus of the funding was to support patient organizations’ educational activities, accounting for 40.4 percent of the total (see Table 3). Such activities included, for example, educational events or establishing patient information hotlines. Project support received 33.0 percent of the funding, while conference sponsorship accounted for 26.6 percent. In 2020, companies dedicated €216 082 (8.9% of the total funding for that year) to support COVID-19-related activities (see Table 3). There were also 123 payments, totaling €761 108 (5.5% of the total), with either uninformative or completely absent payment descriptions.

**Table 3.** Goal of Drug Industry Funding for Patient Organization Projects and Activities in Poland, 2012–2020.

Payment goals	No. of recipients (%)	No. of donors (%)	No. of payments (%)	Median payment (IQR), Euro	Value of payments, Euro (% of total payments)*
Educational activities	157 (57.5%)	29 (87.9%)	956 (36.9%)	3048 (1519 to 5880)	5 541 131 (40.4%)
Project support	123 (45.1%)	24 (72.7%)	548 (21.2%)	4704 (2352 to 7799)	4 525 970 (33.0%)
Sponsorship of conferences, forums, conventions, workshops, training, debates, webinars.	49 (54.5%)	28 (84.8%)	735 (28.4%)	2929 (1514 to 5880)	3 645 283 (26.6%)
Support for publishing books, brochures, leaflets, films (printing, preparation)	64 (23.4%)	23 (69.7%)	249 (9.6%)	3662 (2224 to 5438)	1 130 819 (8.2%)
Support for statutory activities	66 (24.2%)	18 (54.5%)	201 (7.8%)	3576 (1986 to 5959)	994 552 (7.2%)
Website reconstruction or creation, applications, e-learning, radio, internet television, social media	49 (17.9%)	19 (57.6%)	104 (4.0%)	3741 (2019 to 7152)	770 544 (5.6%)
Uninformative description, no description	60 (22.0%)	17 (51.5%)	123 (4.8%)	3376 (1194 to 5177)	761 108 (5.5%)
Activities for patients, free consultations, organization of stays, charitable events	71 (26.0%)	24 (72.7%)	154 (6.0%)	2352 (1191 to 3752)	585 860 (4.3%)
Support for research (e.g., screening tests, diagnostics tests, scientific and social research, research reports, expert opinions)	35 (12.8%)	19 (57.6%)	76 (2.9%)	2565 (1694 to 5712)	420 210 (3.1%)
Public relations, communication activities	13 (4.8%)	10 (30.3%)	26 (1.0%)	4501 (2386 to 9554)	300 066 (2.2%)
Purchasing or providing equipment	48 (17.6%)	19 (57.6%)	84 (3.2%)	2367 (817 to 4614)	283 857 (2.1%)
COVID-19 help*	28 (10.3%)	16 (48.5%)	37 (1.4%)	3376 (1116 to 7878)	216 082 (1.6%)
Other	16 (5.9%)	10 (30.3%)	26 (1.0%)	2899 (497 to 6516)	154 554 (1.1%)
Travel sponsorship for the organization representatives	37 (13.5%)	12 (36.4%)	79 (3.1%)	715 (349 to 1465)	97 910 (0.7%)
Expenses out of place	10 (3.7%)	5 (15.2%)	18 (0.7%)	642 (349 to 2467)	24 359 (0.2%)
Sponsored lecture	13 (4.8%)	8 (24.2%)	17 (0.7%)	470 (246 to 931)	23 895 (0.2%)

IQR: stands for Interquartile Range. * Values will add up to more than 100% as one payment can be in multiple categories.

* Appeared only in 2020.

Of the 273 patient organizations, 148 (54.2%) held the special status of public benefit organizations. These organizations received 66.1 percent (€9 072 087) of the total funding. However, the remaining 125 organizations without the public benefit status received payments with a higher median value of €3594 (interquartile range: €1482 to €7055), while for organizations with the public benefit status, the median value was €2831 (interquartile range: €1410 to €5223). The difference between the two groups was statistically significant (Chi^2 ^= 13.323, *df *= 1, *p *< .001).

Patient organizations based in the Masovian administrative region, where the country's capital, Warsaw, is located, received the largest share of payments. These organizations accounted for the highest payment number (n = 1598; 61.8%) and value (€9 085 131; 66.2%). Furthermore, the Masovian region had the greatest share of patient organizations in our sample (n = 105; 37.2%). The median payment value for organizations in this region was €3662 (with an interquartile range of €2149 to €7144). The differences in the median payment values were statistically significant across the voivodeships, as determined by the Kruskal-Wallis test (H(5) = 143.676, *p *< .01). Specific pairwise comparisons also revealed significant differences (see Online Appendix 9).

Patient organizations with the legal form of foundations, comprising 42.5 percent (n = 116) of the sample total, received the highest payment share of 57.1 percent (€7 834 655) (Online Appendix 9). Conversely, federations (n = 8; 2.9%) received the highest payment values on average, with a median of €4501 (and an interquartile range of €2359 to €6979). The differences in payment medians across patient organizations with different legal forms were statistically significant based on the Kruskal-Wallis test (H(2) = 112.424, *p *< .01). Pairwise comparisons suggest this was due to a lower payment median for associations (see Online Appendix 9).

Regarding the stated aims of the 273 patient organizations (with some organizations having multiple aims, leading to overlapping numbers), the largest funding share went to organizations pursuing broadly understood public involvement (n = 129; 47.6%). These activities encompassed advancing patient interests and rights, engaging in politics, lobbying, and media work. They received 65.4 percent (€8 984 572) of the total funding. This was followed by patient organizations (n = 130; 47.6%) pursuing health promotion and disease prevention, which received funding amounting to 56.4 percent (€7 739 499) of the total. Patient organizations (n = 199; 72.4%) supporting patients directly received 55.0 percent (€7 545 904) of the total. For a detailed breakdown of these funding patterns, see Table 4.

**Table 4.** The Aims of Patient Organizations Funded by Drug Companies in Poland, 2012–2020.

Aim	No. of recipients (%)	No. of donors (%)	No. of payments (%)	Median payment (IQR), Euro	Value of payments, Euro (% of total payments)*
Representing or advancing patient interests or rights, lobbying, media	129 (47.3%)	32 (97.0%)	1763 (68.1%)	3376 (1465 to 6115)	8 984 572 (65.4%)
Health promotion and disease prevention	130 (47.6%)	32 (97.0%)	1314 (50.8%)	2986 (1430 to 6234)	7 739 500 (56.4%)
Supporting patients	200 (73.3%)	31 (93.9%)	1514 (58.5%)	2623 (1236 to 4942)	7 545 904 (55.0%)
Education	138 (50.5%)	33 (100%)	1384 (53.5%)	3376 (1247 to 5959)	6 946 300 (50.6%)
Charity work benefitting patients, social assistance, financial assistance	83 (30.4%)	31 (93.9%)	939 (36.3%)	2858 (1328 to 5953)	4 475 467 (32.6%)
Social integration, bring together patients, organizing events, self-help	87 (31.9%)	28 (84.8%)	971 (37.4%)	2471 (1194 to 4987)	4 308 138 (31.4%)
Addressing social exclusion, promoting employment of patients	46 (16.8%)	26 (78.8%)	750 (29.0%)	2931 (1544 to 5948)	3 285 678 (23.9%)
Improving access to medical services, drugs, equipment, improving care for patients	72 (26.4%)	28 (84.8%)	638 (24.7%)	3218 (1236 to 5880)	3 273 268 (23.8%)
Cooperation with other organizations in the country and abroad	49 (17.9%)	29 (87.9%)	578 (22.3%)	3592 (1746 to 5970)	2 814 781 (20.5%)
Rehabilitation stays, trips	61 (22.3%)	26 (78.8%)	590 (22.8%)	2471 (1236 to 4768)	2 509 709 (18.3%)
Other	41 (15.0%)	24 (72.7%)	383 (14.8%)	2384 (1192 to 4942)	1 776 879 (12.9%)
Research (including medical)	44 (16.1%)	27 (81.8%)	349 (13.5%)	3572 (1755 to 5960)	1 662 460 (12.1%)
Actions related to diagnostics	18 (6.6%)	23 (69.7%)	258 (10.0%)	4056 (2352 to 7164)	1 593 001 (11.6%)
Supporting volunteering	23 (8.4%)	19 (57.6%)	199 (7.7%)	3574 (1380 to 7164)	1 312 823 (9.6%)
Improving patients’ quality of life	31 (11.4%)	21 (63.6%)	268 (10.4%)	2471 (1236 to 4942)	1 241 064 (9.0%)
Helping hospital care	30 (11.0%)	20 (60.6%)	89 (3.4%)	2929 (1582 to 6069)	567 209 (4.1%)
Cooperation with pharmaceutical companies and manufacturers of medical technologies	1 (0.4%)	2 (6.0%)	6 (0.2%)	799 (523 to 2096)	8585 (0.1%)

IQR: stands for Interquartile Range. * Values will add up to more than 100% as one payment can be in multiple categories.

#### Trends Over Time

The analysis of total payments over time is complicated because the number of companies with available disclosure reports rose from 12 in 2012 to 28 in 2018 and 2019, and then to 25 in 2020. Nevertheless, ten companies reported payments in all years, accounting for 52.1 percent (1349) of the payments, totaling €7 879 446 (57.4%) (see Table 5).

**Table 5.** Distribution of Drug Company Payments to Patient Organizations in Poland, 2012–2020: Ten Companies That Disclosed Consistently in all Years.

	2012	2013	2014	2015	2016	2017	2018	2019	2020	Total
No. of payments	148	176	159	146	128	121	147	156	168	1349
Value of payments, Euro (% of all payments)	577 825 (74.5%)	740 498 (84.9%)	851 954 (80.4%)	1 254 500 (77.2%)	922 738 (63.8%)	893 225 (57.4%)	904 147 (44.9%)	718 971 (36.6%)	1 015 588 (41.9%)	7 879 446 (57.4%)
Median (IQR)	1787 (953 to 3574)	2441 (1221 to 4883)	2471 (1433 to 4942)	3117 (1496 to 5937)	3731 (2146 to 7152)	4620 (2015 to 8335)	4108 (1552 to 7203)	3528 (2334 to 5880)	4501 (2251 to 6793)	3528 (1496 to 5970)
No. of patient organizations	69	68	69	75	67	56	54	57	62	185**

IQR: stands for Interquartile Range. *Amgen, AstraZeneca, Biogen, Chiesi, GlaxoSmithKline, Merck, Janssen-Cilag, Pfizer, Roche, Takeda

**Refers to the total number of patient organizations that received funding according to company reports, 2012–2020

Payments made by these ten companies more than doubled between 2012 and 2015, from €577 825 to €1 254 500. However, from 2015 onwards, the payments fluctuated between €718 971 and €1 015 588. The data from these ten companies did not indicate a significant increase in the number of patient organizations receiving funding or the number of reported payments. Instead, the increase in payment value was associated with a trend towards higher-value payments, with a significant increase in the median values for both the ten companies reporting payments in all years (Kendall's tau-b = 0.72; *p *= .007) and all 33 companies combined (Kendall's tau-b = 0.56; *p *= .037).

### Structure of Connections Between Companies and Patient Organizations

Most patient organizations seemed to form ties with only one or a few donors (see Online Appendix 10). Of the 273 patient organizations, 53.8 percent (n = 147) were linked to a single company, while 19 percent (n = 52) had connections with two companies. A notable 12.1 percent (n = 33) had more than five, indicating higher diversity in funding relationships, with the Polish Coalition of Oncological Patients’ Foundation (*Fundacja Polska Koalicja Pacjentów Onkologicznych*), an umbrella organization comprising 44 cancer patient organizations, being the most connected (21 companies).

The connections established through payments were mostly sporadic rather than recurring over multiple years (see Table 6). Notably, 44.9 percent (n = 330) of connections were reported in only one of the study years. An additional 30.5 percent (n = 204) were reported in two or three years. The figures were similar for the ten companies reporting payments in all years, with 42.8 percent (n = 139) occurring in one year and 23.6 percent (n = 106) in two or three years, indicating a transient nature of many financial ties.

**Table 6.** Durability of Collaboration Between Patient Organizations and Pharmaceutical Companies in Poland, 2012–2020.

Years of collaboration	1	2	3	4	5	6	7	8	9
*All companies with available data*									
No. of cases of cooperation between patient organizations and companies	326 (48.9%)	125 (18.8%)	81 (12.2%)	50 (7.5%)	36 (5.4%)	21 (3.2%)	15 (2.3%)	9 (1.4%)	3 (0.5%)
Value of payments, Euro	1 488 238 (10.8%)	1 766 469 (12.9%)	2 001 686 (14.6%)	1 408 453 (10.3%)	1 700 853 (12.4%)	2 760 965 (20.1%)	944 579 (6.9%)	909 280 (6.6%)	749 120 (5.5%)
*10 companies reporting in all years*									
No. of cases of cooperation between patient organizations and companies	139 (42.8%)	59 (18.2%)	47 (14.5%)	30 (9.2%)	19 (5.8%)	13 (4.0%)	9 (2.8%)	6 (1.8%)	3 (0.9%)
Value of payments, Euro	623 528 (7.9%)	492 215 (6.2%)	856 883 (10.9%)	801 910 (10.2%)	974 926 (12.4%)	2 263 339 (28.7%)	633 910 (8.0%)	483 616 (6.1%)	749 120 (9.5%)

Only a small percentage of connections occurred consistently over the nine-year study period (0.4%, n = 3) or in most years (3.5%, n = 24; seven or eight years). For the ten companies reporting payments in all years, the figures were 0.9 percent (n = 3) and 4.6 percent (n = 15), respectively. Despite the limited occurrence of long-lasting connections, the accumulated monetary value of recurring financial transfers was substantial. Over nine years, it accounted for 5.5 percent (for all companies) and 9.5 percent (for the ten companies reporting in all years), while in seven or eight years, it represented 13.5 percent (for all companies) and 14.2 percent (for the ten companies reporting in all years). This indicates enduring and significant connections for a subset of patient organizations.

## Discussion

We offer the first comprehensive analysis of the pattern, evolution, and structure of financial ties between drug companies and patient organizations in a Central European country.

We drew similarities and differences between the patterns identified in Poland and other previously studied countries (Denmark, Sweden, Finland, the United Kingdom, Australia and the United States) in which transparency of payments to patient organizations is similarly governed through the industry's self-regulation (see Online Appendix 11).

In Poland, the number of companies subscribing to the industry code, and thus bound to disclose payments (33), was considerably lower than in Sweden (99), Norway (55), and the United Kingdom (61); it was also slightly lower than in Denmark (36) and Finland (39). These differences may reflect the industry's specific country profiles, including the balance between smaller versus larger companies and those manufacturing generic products versus those focusing on new molecules.^31^ Unlike the United Kingdom and Sweden, Poland does not have a sizeable sector of home-grown pharmaceutical companies, with few smaller start-ups engaged in drug discovery. However, the comparisons are not straightforward. For example, despite having a similar number of trade-group affiliated companies to Poland, Denmark has a significant life science industry, including major global companies (Leo Pharma, Lundbeck and Novo Nordisk).

Absent legally mandated disclosures, we can only compare the minimum extent of drug company funding across the countries with an unknown number of unreported or misreported payments.^11,52,62^ In Poland, the reported yearly totals were €1–2 million. While this pattern was similar to Denmark and Sweden, Poland's population is considerably larger (6 million and 10 million versus 38 million, respectively).^17^ Direct comparison of total funding is further confounded by the differences in the purchasing power parity and gross domestic product per capita. Nevertheless, in Poland, just like elsewhere, a few top donors dominated, largely overlapping with the list of the world's largest “Big Pharma” companies.^61^

Many other payment patterns were also consistent with those reported in other countries. For example, approximately 30 percent of payments were over €5000, placing Poland between Sweden (∼20%)^17^ and Denmark (∼40%).^19^ In addition, the ten largest payments constituted roughly 10 percent of the total value in Poland, compared to 9 percent in Denmark^19^ and 13 percent in Sweden.^17^ In Poland, as in Sweden,^17^ Denmark,^19^ the United Kingdom,^9^ and Australia,^7^ companies were mainly interested in funding cancer patient organizations. Also, similar to Australia,^7^ the greatest share of payments went to education, while in Sweden^17^ it went to communication, and in the United Kingdom^9^ it went to research.

Like in the United Kingdom,^18^ we found funding is concentrated around the country's capital. In our case, the Masovian administrative region can be interpreted as a fertile ground for growing social networks between drug companies and patient organizations. All companies from our sample were headquartered in this voivodeship, which also has the largest density of patient organizations at 2.7 per 10 000 people.^34^ From the companies’ perspective, patient organizations located close to the country's parliament, executive (e.g., the Ministry of Health), and expert advisory bodies (the Agency for Health Technology Assessment) might be the most attractive lobbying allies.

The payment share going to public benefit organizations (66.1% of the total) was unsurprising as these organizations commit, at least in principle, to higher standards of accountability and transparency. By being expected to have appropriate procedures for managing external funding, they could be regarded as more trustworthy and reliable collaborators for drug companies. However, the share of funding received by public benefit organizations was considerably lower than in the case of U.K. charities (94.4%), which are also expected to fulfil distinct transparency requirements^62^ and enjoy tax benefits on donations received.^63^ In Poland, pharmaceutical companies give a larger share of payments to foundations (57.1% of the total), which is the opposite of the United Kingdom^9^; this is difficult to interpret given that foundation rules and parameters may differ for patient organizations in the two countries.

Comparing trends in payment values across countries is challenging given possible gaps in companies’ reporting and the fact that the trends reported in other studies typically cover all available payment data, as opposed to companies reporting their payments consistently and retaining the records in the public domain.^9,19^ Nevertheless, of all the studied countries, Poland represents the clearest example of an upward trend of increasing funding. This likely reflects strong incentives driving access-oriented consumerism^64^ over the last decade. In Poland, significant gross domestic product growth has elevated the statutory threshold of cost effectiveness, a key criterion for recommending drugs for state reimbursement.^42,43^ This was associated with the increasing coverage of new therapies, especially in oncology.^65^ Conversely, collaborations between drug companies and patient organizations can also be triggered by implementing strict cost-saving measures, putting Poland's public pharmaceutical spending far below (37% versus 56%)^66^ and out-of-pocket spending considerably above (12.6% versus 3.7%)^47^ the E.U. average. One additional incentive to collaborate with drug companies may be the democratic backsliding detected in Poland in recent years.^67^ Consequently, multinationals may be seen as funding sources less vulnerable to political control than local businesses or public bodies.^42^

Like in Denmark^19^ and Sweden,^17^ we saw a combination of prevalent short-term financial relationships coupled with a few durable connections. Still, contextualizing the trend to form exclusive—or nearly exclusive—financial ties is difficult. The only available comparator is Denmark,^19^ characterized by the dominance of a single company, which was the sole funder of a sizable subset of patient organizations. The pattern we detected in this study is consistent with the prominent role played in Poland's health policymaking by elite cliques whose members span the state, private, and nongovernmental sectors.^42^ Clique activity has been described as conducive to maintaining financial relationships with only one or a few drug companies.^42–44,68^ This pattern of more exclusive relationships is also consistent with the evidence of widespread corruption and informal practices in Poland's health care sector.^45^

Systematic analysis of the impact of drug company funding on patient organizations should be a research priority in Poland and elsewhere.^15^ It is likely that drug company funding, especially over a longer period, can be a form of disguised marketing. Such funding might impact the information patients receive about treatments, the advocacy efforts of patient groups, their lobbying for the approval and reimbursement of certain drugs, and, more generally, could shift patient groups’ discourse and ideology in ways that align more with corporate than patient interests.^1,24^ Drug company funding can also shape the structure and activities of the patient group sector as a whole by amplifying specific causes and voices over others.^9^ There is also the risk of bias and loss of trust resulting from conflicts of interest, especially in areas such as drug reimbursement and health technology assessment.^10,69^

Our findings reinforce the case for comprehensive sunshine legislation that would apply to all drug companies and recipients of drug company funding. While not without its limitations,^70^ sunshine legislation is superior to industry self-regulation in achieving transparency by mandating comprehensive, high-quality and user-friendly disclosures.^71,72^ At the same time, transparency alone is insufficient to address financial conflicts of interest and industry influence on the health care sector. Thus, additional measures may be warranted, such as excluding patient organizations with a high level of industry funding from certain forms of policy involvement.

### Limitations

Given the possible underreporting of payments and the fact that some companies removed older disclosure reports after the EFPIA minimum three-year data retention period, the true extent of drug company funding is unknown.^11,19,62^ Further, the Infarma members represent only a small share of the approximately 150 pharmaceutical companies operating in Poland. Notably, the generic drug sector—prominent in the number of companies and sales volume^73^—was not considered as they did not sign up to the Infarma Code. Finally, we did not review patient organization websites to corroborate the drug company reports,^14^ which would likely have revealed important discrepancies.^62^

## Conclusion

The pattern of financial ties between drug companies and patient organizations in Poland was, in many key ways, similar to Western countries, including the concentration of funding, the prioritization of cancer patient advocacy, and the prevalent pattern of short-term financial relationships coupled with few durable high-value connections. Certain aspects of the payment distribution were more similar to some countries than others; for example, the emphasis on education activities resembled the patterns observed in Australia more than those in the previously studied European countries. Poland was notable for the increasing value of both the overall funding and individual payments. Poland also displayed a pattern of exclusivity of funding relationships, the uniqueness of which will need to be further examined. Like other studies, ours reinforces the case for state-mandated comprehensive disclosure of industry funding of patient organizations.^3,20,22^

## Supplemental Material

sj-docx-1-joh-10.1177_27551938241305995 - Supplemental material for Pharmaceutical Industry Payments to Patient Organizations in Poland: Analysis of the Patterns, Evolution, and Structure of Connections Supplemental material, sj-docx-1-joh-10.1177_27551938241305995 for Pharmaceutical Industry Payments to Patient Organizations in Poland: Analysis of the Patterns, Evolution, and Structure of Connections by Marta Makowska, Shai Mulinari and Piotr Ozieranski in International Journal of Social Determinants of Health and Health Services

sj-docx-2-joh-10.1177_27551938241305995 - Supplemental material for Pharmaceutical Industry Payments to Patient Organizations in Poland: Analysis of the Patterns, Evolution, and Structure of Connections Supplemental material, sj-docx-2-joh-10.1177_27551938241305995 for Pharmaceutical Industry Payments to Patient Organizations in Poland: Analysis of the Patterns, Evolution, and Structure of Connections by Marta Makowska, Shai Mulinari and Piotr Ozieranski in International Journal of Social Determinants of Health and Health Services

sj-docx-3-joh-10.1177_27551938241305995 - Supplemental material for Pharmaceutical Industry Payments to Patient Organizations in Poland: Analysis of the Patterns, Evolution, and Structure of Connections Supplemental material, sj-docx-3-joh-10.1177_27551938241305995 for Pharmaceutical Industry Payments to Patient Organizations in Poland: Analysis of the Patterns, Evolution, and Structure of Connections by Marta Makowska, Shai Mulinari and Piotr Ozieranski in International Journal of Social Determinants of Health and Health Services

sj-docx-4-joh-10.1177_27551938241305995 - Supplemental material for Pharmaceutical Industry Payments to Patient Organizations in Poland: Analysis of the Patterns, Evolution, and Structure of Connections Supplemental material, sj-docx-4-joh-10.1177_27551938241305995 for Pharmaceutical Industry Payments to Patient Organizations in Poland: Analysis of the Patterns, Evolution, and Structure of Connections by Marta Makowska, Shai Mulinari and Piotr Ozieranski in International Journal of Social Determinants of Health and Health Services

sj-docx-5-joh-10.1177_27551938241305995 - Supplemental material for Pharmaceutical Industry Payments to Patient Organizations in Poland: Analysis of the Patterns, Evolution, and Structure of Connections Supplemental material, sj-docx-5-joh-10.1177_27551938241305995 for Pharmaceutical Industry Payments to Patient Organizations in Poland: Analysis of the Patterns, Evolution, and Structure of Connections by Marta Makowska, Shai Mulinari and Piotr Ozieranski in International Journal of Social Determinants of Health and Health Services

sj-docx-6-joh-10.1177_27551938241305995 - Supplemental material for Pharmaceutical Industry Payments to Patient Organizations in Poland: Analysis of the Patterns, Evolution, and Structure of Connections Supplemental material, sj-docx-6-joh-10.1177_27551938241305995 for Pharmaceutical Industry Payments to Patient Organizations in Poland: Analysis of the Patterns, Evolution, and Structure of Connections by Marta Makowska, Shai Mulinari and Piotr Ozieranski in International Journal of Social Determinants of Health and Health Services

sj-docx-7-joh-10.1177_27551938241305995 - Supplemental material for Pharmaceutical Industry Payments to Patient Organizations in Poland: Analysis of the Patterns, Evolution, and Structure of Connections Supplemental material, sj-docx-7-joh-10.1177_27551938241305995 for Pharmaceutical Industry Payments to Patient Organizations in Poland: Analysis of the Patterns, Evolution, and Structure of Connections by Marta Makowska, Shai Mulinari and Piotr Ozieranski in International Journal of Social Determinants of Health and Health Services

sj-docx-8-joh-10.1177_27551938241305995 - Supplemental material for Pharmaceutical Industry Payments to Patient Organizations in Poland: Analysis of the Patterns, Evolution, and Structure of Connections Supplemental material, sj-docx-8-joh-10.1177_27551938241305995 for Pharmaceutical Industry Payments to Patient Organizations in Poland: Analysis of the Patterns, Evolution, and Structure of Connections by Marta Makowska, Shai Mulinari and Piotr Ozieranski in International Journal of Social Determinants of Health and Health Services

sj-docx-9-joh-10.1177_27551938241305995 - Supplemental material for Pharmaceutical Industry Payments to Patient Organizations in Poland: Analysis of the Patterns, Evolution, and Structure of Connections Supplemental material, sj-docx-9-joh-10.1177_27551938241305995 for Pharmaceutical Industry Payments to Patient Organizations in Poland: Analysis of the Patterns, Evolution, and Structure of Connections by Marta Makowska, Shai Mulinari and Piotr Ozieranski in International Journal of Social Determinants of Health and Health Services

sj-docx-10-joh-10.1177_27551938241305995 - Supplemental material for Pharmaceutical Industry Payments to Patient Organizations in Poland: Analysis of the Patterns, Evolution, and Structure of Connections Supplemental material, sj-docx-10-joh-10.1177_27551938241305995 for Pharmaceutical Industry Payments to Patient Organizations in Poland: Analysis of the Patterns, Evolution, and Structure of Connections by Marta Makowska, Shai Mulinari and Piotr Ozieranski in International Journal of Social Determinants of Health and Health Services

sj-docx-11-joh-10.1177_27551938241305995 - Supplemental material for Pharmaceutical Industry Payments to Patient Organizations in Poland: Analysis of the Patterns, Evolution, and Structure of Connections Supplemental material, sj-docx-11-joh-10.1177_27551938241305995 for Pharmaceutical Industry Payments to Patient Organizations in Poland: Analysis of the Patterns, Evolution, and Structure of Connections by Marta Makowska, Shai Mulinari and Piotr Ozieranski in International Journal of Social Determinants of Health and Health Services
