# Supplementary material for: Pharmaceutical Industry Payments to Patient Organizations in Poland: Analysis of the Patterns, Evolution, and Structure of Connections
Source: Int J Soc Determinants Health Health Serv. 2024 Dec 26;55(2):199–212. doi: 10.1177/27551938241305995 (PMC11977834; doi:10.1177/27551938241305995)
Supplement: sj-docx-1-joh-10.1177_27551938241305995 - Supplemental material for Pharmaceutical Industry Payments to Patient Organizations in Poland: Analysis of the Patterns, Evolution, and Structure of Connections [file sj-docx-1-joh-10.1177_27551938241305995.docx]

*Appendix 1. Main differences between foundations, associations and federations*

|  | ***Foundation*** | ***Association*** | ***Federation*** |
| --- | --- | --- | --- |
| *Purpose* | *It is a non-governmental organization that operates to achieve the goal set by the founder or the foundation's board. It raises funds to allocate them to a socially important goal.* | *An association is a group of people who want to develop their own interests and achieve goals.* | *It is a union of associations. It brings together organizations that cooperate for a specific purpose. A foundation can also join a federation.* |
| *Establishment* | \|  \| \| --- \|  \| *Founder* \| \| --- \| | *Minimum 7 people* | *3 associations* |
| *Need for initial funds* | *Min. 220 Euro* | *No* | *No* |
| *Membership fees* | *No* | *Yes* | *Yes* |
| *Legal personality (registered in KRS)* | *Yes* | *Yes* | *Yes* |
| *Possibility to conduct business activities* | *Yes* | *Yes* | *Yes* |
