# Supplementary material for: Pharmaceutical Industry Payments to Patient Organizations in Poland: Analysis of the Patterns, Evolution, and Structure of Connections
Source: Int J Soc Determinants Health Health Serv. 2024 Dec 26;55(2):199–212. doi: 10.1177/27551938241305995 (PMC11977834; doi:10.1177/27551938241305995)
Supplement: sj-docx-2-joh-10.1177_27551938241305995 - Supplemental material for Pharmaceutical Industry Payments to Patient Organizations in Poland: Analysis of the Patterns, Evolution, and Structure of Connections [file sj-docx-2-joh-10.1177_27551938241305995.docx]

Appendix 2. Flowchart of the data collection process

Number of companies with all reports avaiable 2008-2020
(n=3)

Number of reports (n_r_) and payments (n_p_) from 2008-2020 identified during searching 33 Infarma's members (past and present) web pages
(n_r_=202; n_p_=3170)

Number of exluded reports (n_r_) and exluded payments (n_p_) from 2008-2011 due to small number
(n_r_=19; n_p_=332)

Number of exluded payments (n_p_) because of inproper recipient
(n_p_=242)

Number of reports excluded because of lack of payments
(n_r_=6)

Number of companies with all raports avaiable 2011-2020
(n=10)

Final number of reports (n_r_) and payments (n_p_) from 2011-2020 identified during searching 33 Infarma's members (past and present) web pages
(n_r_=183; n_p_=2596)
