# Supplementary material for: Pharmaceutical Industry Payments to Patient Organizations in Poland: Analysis of the Patterns, Evolution, and Structure of Connections
Source: Int J Soc Determinants Health Health Serv. 2024 Dec 26;55(2):199–212. doi: 10.1177/27551938241305995 (PMC11977834; doi:10.1177/27551938241305995)
Supplement: sj-docx-3-joh-10.1177_27551938241305995 - Supplemental material for Pharmaceutical Industry Payments to Patient Organizations in Poland: Analysis of the Patterns, Evolution, and Structure of Connections [file sj-docx-3-joh-10.1177_27551938241305995.docx]

Appendix 3. Recipients excluded from analysis

Recipients who were not recognized n=87 (36.0% of all excluded) value of the payments €311,630 (32.0% of all excluded)

Recipient nature unclear n=29 (12.0% of all excluded) value of the payments €177,896 (18.3% of all excluded)

Non-research country organizations n=8 (3.3% of all excluded) value of the payments €36,428 (3.7% of all excluded)

Payments to orgnisations that focus on public health n=36 (14.8% of all excluded) value of the payments €159,105 (16.4% of all excluded)

Payments to healthcare providers organization n=26 (10.7% of all excluded) €101,197 (10.4% of all excluded)

Payments to hospice n=22 (9.1% of all excluded) value of the payments €31,764 (3.3% of all excluded)

Payments to physicians organisations n=30 (12.4% of all excluded) value of the payments €115,511 (11.9% of all excluded)

Payments to third-sector organisations not focusing directly on health (child care home, trade union) n=4 (1.6% of all excluded) value of the payments €37,372 (3.8% of all excluded)

Recipient is not patient organization (eg. child care home, hospice, trade union, healthcare provider, professional organizations, focus on public health organizations) n=118 (49.0 % of all excluded) value of the payments €444,949 (46.0 % of all excluded)

Payments excluded 2012 -2020 n=242 (8.5%)

due to different reasons,

value of the payments €970,902 (6.6%*) (including 3 payments -  nonfinancial, wrong).
