# Supplementary material for: Pharmaceutical Industry Payments to Patient Organizations in Poland: Analysis of the Patterns, Evolution, and Structure of Connections
Source: Int J Soc Determinants Health Health Serv. 2024 Dec 26;55(2):199–212. doi: 10.1177/27551938241305995 (PMC11977834; doi:10.1177/27551938241305995)
Supplement: sj-docx-4-joh-10.1177_27551938241305995 - Supplemental material for Pharmaceutical Industry Payments to Patient Organizations in Poland: Analysis of the Patterns, Evolution, and Structure of Connections [file sj-docx-4-joh-10.1177_27551938241305995.docx]

| **ICD-10* (previous UK^12,20^ and Swedish**^19^ **studies)** | **Inductive coding** (Poland) |
| --- | --- |
| **I** Certain infectious and parasitic diseases | **Bacterial diseases** |
| **II** Neoplasms | **Oncology** |
| **III** Diseases of the blood and blood-forming organs and certain disorders involving the immune mechanism | **Haematology** |
| **IV** Endocrine, nutritional and metabolic diseases | **Diabetology** |
| **V** Mental and behavioural disorders | **Mental health** |
| **VI** Diseases of the nervous system | **Neurology** |
| **VII** Diseases of the eye and adnexa | **Ophthalmology** |
| **VIII** Diseases of the ear and mastoid process | **NONE** |
| **IX** Diseases of the circulatory system | **Cardiology** |
| **X** Diseases of the respiratory system | **Pulmonology** |
| **XI** Diseases of the digestive system | **Gastroenterology** |
| **XII** Diseases of the skin and subcutaneous tissue | **Dermatology** |
| **XIII** Diseases of the musculoskeletal system and connective tissue | **Rheumatology** |
| **XIV** Diseases of the genitourinary system | **Urology** |
| **XV** Pregnancy, childbirth and the puerperium | **Genecology** |
| **XVI** Certain conditions originating in the perinatal period | **NONE** |
| **XVII** Congenital malformations, deformations and chromosomal abnormalities |  |
| **XVIII** Symptoms, signs and abnormal clinical and laboratory findings, not elsewhere classified |  |
| **XIX** Injury, poisoning and certain other consequences of external causes |  |
| **XX** External causes of morbidity and mortality |  |
| **XXI** Factors influencing health status and contact with health services |  |
| **XXII** Codes for special purposes | **Others** |
| No equivalents | **Patient rights (health education/health promotion)** |
|  | **HIV/AIDS** |
|  | **Rare disease** |
|  | **Hepatology** |
|  | **Nephrology** |
|  | **Transplantology** |
|  | **Immunology** |

Appendix 4. ICD-10 vs. inductive coding of patient organisation disease areas

*Classification in accordance with Terminology and Classifications Delivery Service NHS England, *National Clinical Coding Standards ICD-10 5th Edition;* 2023. Accessed June 30, 2023. https://classbrowser.nhs.uk/ref_books/ICD-10_2023_5th_Ed_NCCS.pdf
