# Supplementary material for: Pharmaceutical Industry Payments to Patient Organizations in Poland: Analysis of the Patterns, Evolution, and Structure of Connections
Source: Int J Soc Determinants Health Health Serv. 2024 Dec 26;55(2):199–212. doi: 10.1177/27551938241305995 (PMC11977834; doi:10.1177/27551938241305995)
Supplement: sj-docx-5-joh-10.1177_27551938241305995 - Supplemental material for Pharmaceutical Industry Payments to Patient Organizations in Poland: Analysis of the Patterns, Evolution, and Structure of Connections [file sj-docx-5-joh-10.1177_27551938241305995.docx]

Appendix 5 – Categorisation of payment goals

| **Previous coding (UK) - single coding ^12.20^** | **Inductive coding (Poland) – multiple coding** |
| --- | --- |
| Accessing organisation's expertise to facilitate drug company activities | N/A |
| Advocacy, campaigning and disease awareness | N/A |
| Communication - media, meetings, online, publications | Public relations, communication activities |
| Education and training | Educational activities |
| Education services provided by drug companies to patient organisations | N/A |
| Funding for awards | N/A |
| Inputting to organisation's work via corporate membership, partnership, sponsorship or support | N/A |
| Organisational maintenance and development, including patient and volunteer engagement | N/A |
| Patient support | Activities for patients, free consultations, organisation of stays, charitable events |
| Policy engagement | N/A |
| Project or programme funding (no specific goals stated) | Project support |
| Research | Support for research (e.g. screening tests, diagnostics tests, scientific and social research, research reports, expert opinions) |
| Support for fundraising with no mention of how the money should be spent | N/A |
| Support for work and activity | Support for statutory activities |
| Lack of information | Uninformative description, no description |
| More than one distinct purpose mentioned | N/A |
| Goal of funding unclear | N/A |
| N/A | Sponsorship of conferences, forums, conventions, workshops, training, debates, webinars. |
| N/A | Expenses out of place |
| N/A | Covid-19 help |
| N/A | Sponsored lecture |
| N/A | Website reconstruction or creation, applications, e-learning, radio, Internet television, social media |
| N/A | Travel sponsorship for the organisation representatives |
| N/A | Purchasing or providing equipment |
| N/A | Support for publishing books, brochures, leaflets, films (printing, preparation) |
| N/A | Other |
