# Supplementary material for: Pharmaceutical Industry Payments to Patient Organizations in Poland: Analysis of the Patterns, Evolution, and Structure of Connections
Source: Int J Soc Determinants Health Health Serv. 2024 Dec 26;55(2):199–212. doi: 10.1177/27551938241305995 (PMC11977834; doi:10.1177/27551938241305995)
Supplement: sj-docx-6-joh-10.1177_27551938241305995 - Supplemental material for Pharmaceutical Industry Payments to Patient Organizations in Poland: Analysis of the Patterns, Evolution, and Structure of Connections [file sj-docx-6-joh-10.1177_27551938241305995.docx]

Appendix 6. Categorisation of patient organisation’s goals

| **Previous coding (UK^12,20^) – single coding** | **Inductive coding (Poland) – multiple coding** |
| --- | --- |
| Advocacy organisation | Representing or advancing patient interests or rights, lobbying, media |
| N/A | Improving patients’ quality of life |
| Organisation focused on education | Education |
| Organisation focused on funding medical research | Research (including medical) |
| Organisation focused on providing funding or material support to NHS organisations or patients | Helping hospital care |
| N/A | Cooperation with other organisations in the country and abroad |
| Support organisation | Supporting patients |
| N/A | Improving access to medical services, drugs, equipment, improving care for patients |
|  | Supporting volunteering |
|  | Health promotion and disease prevention |
|  | Charity work benefitting patients, social assistance, financial assistance |
|  | Social integration, bring together patients, organizing events, self-help |
|  | Rehabilitation stays, trips |
|  | Addressing social exclusion, promoting employment of patients |
|  | Actions related to diagnostics |
|  | Cooperation with pharmaceutical companies and manufacturers of medical technologies |
|  | Other |
| Organisation focusing on funding grants | N/A |
| Multipurpose organisation |  |
