# Supplementary material for: Pharmaceutical Industry Payments to Patient Organizations in Poland: Analysis of the Patterns, Evolution, and Structure of Connections
Source: Int J Soc Determinants Health Health Serv. 2024 Dec 26;55(2):199–212. doi: 10.1177/27551938241305995 (PMC11977834; doi:10.1177/27551938241305995)
Supplement: sj-docx-7-joh-10.1177_27551938241305995 - Supplemental material for Pharmaceutical Industry Payments to Patient Organizations in Poland: Analysis of the Patterns, Evolution, and Structure of Connections [file sj-docx-7-joh-10.1177_27551938241305995.docx]

Appendix 7 – Ten top donors.

| Drug company | Number of payments n (%) | Number of patient organisations funded | Median payment (IQR), Euro | Value of payments, Euro (% of total payments) |
| --- | --- | --- | --- | --- |
| Roche | 531 | 101 | 2,471 (1236 to 4768) | 1,989,651 (14.5%) |
| Pfizer | 114 | 32 | 4,704 (2251 to 11 910) | 1,916,112 (14.0%) |
| Janssen - Cilag | 240 | 58 | 4,765 (2359 to 7413) | 1,629,490 (11.9%) |
| Biogen | 173 | 15 | 4,395 (1963 to 7152) | 1,102,558 (8.0%) |
| Novartis | 251 | 46 | 2,388 (1192 to 4776) | 971,323 (7.1%) |
| Bayer | 243 | 41 | 2,388 (1176 to 4942) | 955,182 (7.0%) |
| Sanofi | 116 | 34 | 4,501 (2352 to 9408) | 759,901 (5.5%) |
| AbbVie | 143 | 41 | 3,376 (1176 to 6191) | 648,309 (4.7%) |
| BMS | 96 | 23 | 3,707 (2385 to 6732) | 535,398 (3.9%) |
| MSD | 58 | 15 | 2,352 (1194 to 3582) | 351,508 (2.6%) |
| Total | 1,965 | Mean 40.6 (SD=25.2) | 3,376 (1483 to 5970) | 10,859,432 (79,1%) |
