# Supplementary material for: Pharmaceutical Industry Payments to Patient Organizations in Poland: Analysis of the Patterns, Evolution, and Structure of Connections
Source: Int J Soc Determinants Health Health Serv. 2024 Dec 26;55(2):199–212. doi: 10.1177/27551938241305995 (PMC11977834; doi:10.1177/27551938241305995)
Supplement: sj-docx-8-joh-10.1177_27551938241305995 - Supplemental material for Pharmaceutical Industry Payments to Patient Organizations in Poland: Analysis of the Patterns, Evolution, and Structure of Connections [file sj-docx-8-joh-10.1177_27551938241305995.docx]

Appendix 8 - Top ten recipients

| Patient organisation name | Condition | Number of payments n (%) | Number of donors | Main funder, Euro, % of all that patient organisation get, years founded by main funder | Years of cooperation, Mean; min, max | Median payment (IQR), Euro | Value of payments, Euro (% of total payments) |
| --- | --- | --- | --- | --- | --- | --- | --- |
| Fundacja "Aby Żyć" | Bacterial diseases (meningococci, pneumococci/infectious/promotion of vaccination) | 12 (0.5%) | 1 | Pfizer, 1,123,812  100%; 6 | 6;6-6 | 57,999 (11,624 to 167,927) | 1,123,812 (8.1%) |
| ,Polskie Towarzystwo Stwardnienia Rozsianego | Neurology | 223 (8.6%) | 7 | Biogen,  464,530; 43.1%;9 | 5.3; 2-9 | 2,471 (1,372 to 7,056) | 1,076,807 (7.8%) |
| Alivia Onkofundacja | Oncology | 82 (3.2%) | 17 | MSD;  181,667; 26.6%; 2 | 2.5;1-6 | 4,769 (2,352 to 9,566) | 683,908 (5.0%) |
| Fundacja Instytut Praw Pacjenta i Edukacji Zdrowotnej | Patient rights & health education | 59 (2.3%) | 10 | Janssen – Cilag;  316,594; 46.9%; 6 | 3.4;1-7 | 7,413 (4,776 to 12,356) | 674,686 (4.9%) |
| Fundacja Polska Koalicja Pacjentów Onkologicznych | Oncology | 165 (6.4%) | 21 | Roche; 146,151; 23.4%; 9 | 3.6; 1-9 | 3,528 (2,167 to 4,772) | 624,588 (4.5%) |
| Krajowe Forum na rzecz terapii chorób rzadkich ORPHAN | Rare Disease | 80 (3.1%) | 15 | Sanofi;  193,262; 36.5%; 3 | 2.9;1-6 | 5,627 (3,649 to 7,878) | 529,839 (3.9%) |
| Fundacja Urszuli Jaworskiej | Oncology | 38 (1.5%) | 9 | Biogen,  168,174; 38.2%; 7 | 2.8; 1-7 | 6,069 (3,523 to 8,834) | 440,480 (3.2%) |
| Fundacja SM - Walcz o Siebie | Neurology | 50 (1.9%) | 6 | Biogen; 134,108; 31.0%; 4 | 4; 3-5 | 5,786 (2,352 to 12,070) | 432,384 (3.1%) |
| Fundacja Neuropozytywni | Neurology | 62 (2.4%) | 11 | Biogen;  163,414; 42.2%;8 | 3.1; 1-8 | 4,632 (2,387 to 7,481) | 386,990 (2.8%) |
| Fundacja Carita im. Wiesławy Adamiec | Neoplasms   (myeloma) | 38 (1.5%) | 5 | Janssen – Cilag; 205,098; 56.2%; 6 | 2.4; 1-6 | 6,316 (2,547 to 12,395) | 364,639 (2.7%) |
| TOTAL | N/A | 809 (31.3%) | 26 | Pfizer  1,383,638 21.3%* | N/A | 4,489 (2,251 to 7,562) | 6,338,133  (46.2%) |
