# Supplementary material for: Pharmaceutical Industry Payments to Patient Organizations in Poland: Analysis of the Patterns, Evolution, and Structure of Connections
Source: Int J Soc Determinants Health Health Serv. 2024 Dec 26;55(2):199–212. doi: 10.1177/27551938241305995 (PMC11977834; doi:10.1177/27551938241305995)
Supplement: sj-docx-9-joh-10.1177_27551938241305995 - Supplemental material for Pharmaceutical Industry Payments to Patient Organizations in Poland: Analysis of the Patterns, Evolution, and Structure of Connections [file sj-docx-9-joh-10.1177_27551938241305995.docx]

Appendix 9 - Regions and legal form of patient organisations

| **Regions** | | | | |
| --- | --- | --- | --- | --- |
| Name (no. of patient organisations*) | No of payments | Value of payments (% of all) | Median (IQR), Euro | Kruskall- Wallis mean rank |
| Masovian (105) | 1,599 | 9,085,131 (66.2%) | 3,662 (2,149 to 7,144) | 1424.95 |
| Lesser Poland (26) | 154 | 1,785,833 (13%) | 2,949 (1,244 to 5,241) | 1276.07 |
| Lower Silesia (20) | 99 | 503,596 (3.7%) | 2,251 (977 to 5,218) | 1137.58 |
| Greater Poland (19) | 125 | 385,313 (2.8%) | 2,388 (1,191 to 3,741) | 1065.33 |
| Silesia  (16) | 122 | 375,640 (2.7%) | 2,386 (1,247 to 4,501) | 1119.97 |
| Other 10. (96) | 489 | 1,593,005 (11.6%) | 2,197 (1,014 to 3,741) | 1007.65 |

| **Bonferroni correction for multiple comparisons of Kruskal Wallis test** | | | | |
| --- | --- | --- | --- | --- |
| Name (no. of recipients) | Test Statistic | Std. Error | Std. Test Statistic | P** |
| Masovian - Lesser Poland | 148,878 | 63,045 | 2,361 | .273 |
| Masovian - Lower Silesia | 287,365 | 77,387 | 3,713 | .003 |
| Masovian - Greater Poland | 359,618 | 69,395 | 5,182 | .000 |
| Masovian -Silesia | 304,975 | 70,182 | 4,345 | .000 |
| Masovian -Other | 417,299 | 38,613 | 10,807 | .000 |
| Lesser Poland  - Lower Silesia | 138,487 | 96,255 | 1,439 | 1,000 |
| Lesser Poland  - Greater Poland | 210,740 | 89,956 | 2,343 | .287 |
| Lesser Poland - Silesia | -156,097 | 90,564 | -1,724 | 1,000 |
| Lesser Poland  -Other | 268,421 | 69,045 | 3,888 | .002 |
| Greater Poland - Silesia | 54,643 | 95,094 | ,575 | 1,000 |
| Greater Poland - Lower Silesia | 72,253 | 100,529 | ,719 | 1,000 |
| Greater Poland - Other | 57,681 | 74,889 | ,770 | 1,000 |
| Silesia  - Lower Silesia | -17,609 | 101,074 | -,174 | 1,000 |
| Silesia  - Other | 112,324 | 75,618 | 1,485 | 1,000 |
| Lower Silesia - Other | 129,934 | 82,349 | 1,578 | 1,000 |

| **Legal form** | | | | |
| --- | --- | --- | --- | --- |
| Name (no. of recipients) | No of payments | Value of payments (% of all) | Median (IQR); | Kruskall- Wallis mean rank |
| Foundation (116) | 1,153 | 7,834,655 (57.1%) | 3,707 (2,251 to 7,152) | 1,435.74 |
| Federation, union of associations (8) | 196 | 1,012,542 (7.4%) | 4,501 (2,359 to 6,979) | 1,487.22 |
| Associations (149) | 1,239 | 4,882,447 (35.6%) | 2,406 (1,191 to 4,704) | 1,132.57 |

| **Bonferroni correction for multiple comparisons of Kruskal Wallis test** | | | | |
| --- | --- | --- | --- | --- |
| Name | Test Statistic | Std. Error | Std. Test Statistic | p* |
| Associations - Foundation | 303,170 | 30,575 | 9,915 | .000 |
| Associations - Federation, union of associations | 354,648 | 57,439 | 6,174 | .000 |
| Foundation - Federation, union of associations | -51,479 | 57,730 | -,892 | 1,000 |

* Mind the no of patient organisations won’t sum up to 273, as sometimes there were a branches of the same patient organisations in different regions.

* Significance values have been adjusted by the Bonferroni correction for multiple tests
