# Supplementary material for: Pharmaceutical Industry Payments to Patient Organizations in Poland: Analysis of the Patterns, Evolution, and Structure of Connections
Source: Int J Soc Determinants Health Health Serv. 2024 Dec 26;55(2):199–212. doi: 10.1177/27551938241305995 (PMC11977834; doi:10.1177/27551938241305995)
Supplement: sj-docx-10-joh-10.1177_27551938241305995 - Supplemental material for Pharmaceutical Industry Payments to Patient Organizations in Poland: Analysis of the Patterns, Evolution, and Structure of Connections [file sj-docx-10-joh-10.1177_27551938241305995.docx]

Appendix 10. Number of donors per patient organisation in Poland, 2012-2020

| No. of donors | 2012 | 2013 | 2014 | 2015 | 2016 | 2017 | 2018 | 2019 | 2020 | All years |
| --- | --- | --- | --- | --- | --- | --- | --- | --- | --- | --- |
| 1 (just one payment) | 54 (41) | 51 (41) | 53 (36) | 55 (47) | 58 (49) | 60 (57) | 53 (46) | 51 (40) | 64 (51) | 147 (101) |
| 2 | 19 | 16 | 16 | 21 | 21 | 15 | 16 | 19 | 22 | 52 |
| 3 | 6 | 10 | 6 | 8 | 8 | 13 | 6 | 7 | 8 | 25 |
| 4 | 1 | 3 | 2 | 4 | 4 | 3 | 6 | 6 | 4 | 16 |
| 5 and more | 0 | 0 | 3 | 2 | 4 | 7 | 11 | 12 | 13 | 33 |
| Total | 80 | 80 | 80 | 90 | 95 | 98 | 92 | 95 | 111 | 273 |
