# Supplementary material for: Pharmaceutical Industry Payments to Patient Organizations in Poland: Analysis of the Patterns, Evolution, and Structure of Connections
Source: Int J Soc Determinants Health Health Serv. 2024 Dec 26;55(2):199–212. doi: 10.1177/27551938241305995 (PMC11977834; doi:10.1177/27551938241305995)
Supplement: sj-docx-11-joh-10.1177_27551938241305995 - Supplemental material for Pharmaceutical Industry Payments to Patient Organizations in Poland: Analysis of the Patterns, Evolution, and Structure of Connections [file sj-docx-11-joh-10.1177_27551938241305995.docx]

Appendix 11. Comparable published data on payments from the industry to patient organisations in different countries

|  | **European countries** | | | | **Non-European countries** | |
| --- | --- | --- | --- | --- | --- | --- |
| **Selected characteristics** | **Denmark** | **Poland** | **Sweden** | **UK** | **Australia** | **US** |
| Source | ^21^ | This study | ^19^ | ^12^ | ^10^ | ^30^ |
| Period of observation | 2014-19 | 2012-20 | 2014-18 | 2012-16 | 2013-16 | 2015 |
| Population size | ~ 6m | ~ 38m | ~ 10m | ~ 65m | ~ 26m | ~332m |
| Value of payments, € | ~ €8.8m | ~ €13.7m | ~ €6.4m | ~ €65.1m | ~€23.7m | ~€146.6 m |
| Total no. of payments | 1,224 | 2,588 | 1,337 | 4,572 | 1,487 | N/A |
| Median (IQR) | N/A | 3,105 (1,430 to 5,953) | 2,411 (1,024 to 4,569) | 5,112 (686-11,984) | ~€6,877 (IQR  ~€2,063 –~€17,173) | N/A |
| No. of donor companies | 51 | 33 | 46 | 64 | 34 | 26 |
| No. of patient organisations receiving funding | 84 | 273 | 77 | 508 | 230 | 650 |
| Funding by top ten companies | 75% | 79% | 68% | 69% | 68% | 91% |
| Funding to top ten recipients | 58% | 46% | 62% | N/A | 45% | 59% |
| Top three funding goals (% of value of payments | N/A | Educational actions (40%) | Communication (32%) | Research (25%) | Information / information materials (39%) | N/A |
|  | N/A | Project support (33%) | Advocacy (27%) | Advocacy (14%) | Program and project (39%) | N/A |
|  | N/A | Sponsorship of conferences (27%) | Education (11%) | Communication (12%) | Disease awareness (34%) | N/A |
| Top three funded condition areas | Cancer (25.2%) | Cancer  (38%) | Cancer (38%) | Cancer (38%) | Cancer (21%) | N/A |
|  | Skin and subcutaneous tissue diseases (17%) | Neurology (18%) | Endocrine, nutritional, and metabolic diseases (11%) | Certain infectious and parasitic diseases (8%) | Eye health (13%) | N/A |
|  | Nervous system (11%) | Bacterial diseases (10%) | Certain infectious and parasitic diseases (8%) | Endocrine, nutritional, and metabolic diseases (9%) | Nervous system (10%) | N/A |
| Dominant organisational form | N/A | Public benefit organisations (54.2%) | N/A | Charities  (87.4%) | N/A | N/A |
| Dominant legal forms | N/A | Associations  (54.6%) | N/A | Associations  (34.1%) | N/A | N/A |
